# Lower placental 25-hydroxyvitamin D_3_ (25(OH)D_3_) and higher placental CYP27B1 and 25(OH)D_3_ ratio in preterm birth

**DOI:** 10.1017/jns.2020.42

**Published:** 2020-11-11

**Authors:** Rima Irwinda, Biancha Andardi

**Affiliations:** 1 Maternal-Fetal Medicine, Department of Obstetrics and Gynecology, Faculty of Medicine, Universitas Indonesia, Cipto Mangunkusumo Hospital, Jakarta, Indonesia; 2 Department Obstetrics and Gynecology, Faculty of Medicine, Universitas Indonesia, CIpto Mangunkusumo Hospital, Jakarta, Indonesia

**Keywords:** Preterm birth, 25(OH)D_3_, 1,25(OH)_2_D_3_, CYP27B1

## Abstract

Neonatal mortality rates in Indonesia are still at an alarming rate, with preterm birth as one of the causes. Nutritional deficiencies such as low level of vitamin D is suspected to be the risk factors of preterm birth but still a little knowledge about it. Vitamin D metabolism includes 25-hydroxyvitamin D_3_ (25(OH)D_3_) and 1,25-dihydroxyvitamin D_3_ (1,25(OH)_2_D_3_), as the inactive and active form, with the help of 1α-hydroxylase (CYP27B1) enzyme. Our study aims to determine the differences of 25(OH)D_3_, 1,25(OH)_2_D_3_ and CYP27B1 enzyme in term and preterm birth. A cross-sectional study was performed in Cipto Mangunkusumo National General Hospital, Jakarta, Indonesia, in January–June 2017. The blood sample was taken soon after delivery, to examine maternal 25(OH)D_3_ and 1,25(OH)_2_D_3_ in serum and tissue placenta, as well as placental CYP27B1 enzyme. Statistical analysis using SPPS version 20 was used to find significances. There were a total of sixty subjects in this study, with term-preterm birth group ratio 1:1. We found that placental 25(OH)D_3_ was significantly low (*P* = 0⋅001), and CYP27B1/25(OH)D_3_ ratio was high in preterm birth. Also, there were significant negative correlations found in CYP27B1 level and both placental 25(OH)D_3_ (*r* 0⋅481, *P* < 0⋅001) and 1,25(OH)_2_D_3_ (*r* −0⋅365, *P* = 0⋅004) levels. Our study concludes that preterm birth showed lower placental 25(OH)D_3_ status, and higher CYP27B1/25(OH)D_3_ ratio compared to term pregnancy.

## Introduction

Neonatal mortality is one of the health parameters as well as a determinant of a country's health services. In 2015, it was estimated that the neonatal mortality rate in Indonesia was 14 per 1000 live births, with 35⋅5 % of it is caused by complications of preterm birth^(1)^. Indonesia is ranked eighth out of ten countries with the highest number of neonatal deaths in the world, where there are 66 000 neonatal deaths or 2 % of all neonatal deaths in the world^(1,2)^. Indonesia is also the fifth country with the greatest number of preterm birth, only below India, China, Nigeria and Pakistan^(2)^.

Etiology of preterm birth has not firmly established yet. From the clinical point of view, preterm birth can be caused by maternal, fetal and placental factors. Meanwhile, from the mechanism underlying preterm birth, there are several pathologies associated with preterm birth such as infection, multiple deliveries, genetic predisposition, environmental toxins, intra-amniotic inflammation, fetal allergies, uteroplacental ischaemia, uterine haemorrhage, oxidative stress, excessive uterine distension, immunity factors and nutritional deficiencies^(3)^.

An enormous amount of micro and macronutrients are essential during pregnancy, such as folic acid, iron, zinc, selenium, copper, Vitamin A, Vitamin B, Vitamin C, Vitamin D and Vitamin E^(4,5)^. Some of the nutrients were observed to have a significant correlation with preterm birth incidence, such as folic acid, zinc and Vitamin D. A study by Thota *et al.* showed that Vitamin D has an anti-inflammatory response to inhibit myometrial contractions in the process of preterm birth^(6)^. Furthermore, a research conducted by Tamblyn *et al.* found that vitamin D has a role as an immunomodulatory and antibacterial secretion aggregator during pregnancy through the natural immune system and is obtained in pregnant women^(7)^. Another study by Irwinda *et al.* also showed that preterm birth mother had significantly lower micronutrients such as AtRA, manganese, copper, zinc, iron, copper, selenium and vitamin D^(8)^.

Not only to transfer the nutrients but placenta also plays an important role as a link between mother and fetus by forming decidua. This decidua will act as a place for the presence of various immune cells during pregnancy, including Vitamin D^(9)^. In vitamin D metabolism, it first undergoes hydroxylation by the enzyme 25-Hydroxylase to form 25-Hydroxyvitamin D_3_ (25(OH)D_3_). Then, 25(OH)D_3_, which is the main form of vitamin D in the maternal circulation, will be carried by vitamin D binding protein to the kidneys and placenta. In the kidneys, with the help of the enzyme 1α-hydroxylase (CYP27B1) will form an active form of vitamin D, namely 1,25-Dihidroxyvitamin D_3_ (1,25(OH)_2_D_3_). In the placenta, CYP27B1 and vitamin D receptors (VDR) are expressed in order to extra-renally synthesise 1,25(OH)_2_D_3_^(9)^. This data suggest that placenta has a role in synthesising vitamin D.

In a previous systematic review of the status of Vitamin D globally in 2015, a prevalence of 54 % of pregnant women in deficiency was found, and 18 % in severe deficiency^(10)^. Moreover, a study by Wibowo and Irwinda in Jakarta, Indonesia, also showed a deficiency of Vitamin D level during the first trimester in 99 % of the population^(6)^. Wei *et al.* also conducted a systematic review and meta-analysis of the relationship between Vitamin D status and the incidence of preterm birth, and it was found that Vitamin D deficiency status was a risk factor for preterm birth^(11)^.

This study aims to determine the status of Vitamin D derivate, which are 25(OH)D_3_, and 1,25(OH)_2_D_3_ in maternal serum and placenta, and its regulation in placenta by CYP27B1 between term and preterm birth.

## Methods

This is an analytic observational study using the cross-sectional method to assess the status of 25(OH)D_3_, 1,25(OH)_2_D_3_ in maternal serum and placental tissue, and placental CYP27B1 enzyme between term and preterm birth. Data were taken from Cipto Mangunkusumo Hospital, Jakarta, Indonesia, from January 2017 to August 2019. Using the random sampling method, thirty normal pregnancy and thirty preterm birth samples were used in this study.

The inclusion criteria for the study were a mother with a single intrauterine pregnancy, whether having preterm or term pregnancy. Mothers with multiple pregnancy, fetal growth restriction, congenital anomaly, preterm premature rupture of membrane (PPROM) or having other systemic comorbidities were excluded in this study.

Maternal blood and placental tissue samples were directly taken after delivery. Sample with delivery of more than 1 h will not be included. In order to acquire the status of the 25(OH)D_3_ and 1,25(OH)_2_D_3_ level, a *liquid chromatography-tandem mass spectrometry* (LCMS/MS) method was used. This assay demonstrated good intra and interassay precision, with CV <10 %. An *Agilent 6460 triplequad LCMS system* was used to measure 25(OH)D_3_ and *Acquity I Class Binary Solvent Manager FTN* and *Xevo TQXS Tandem Mass Spectrometry* for 1,25(OH)_2_D_3_. Furthermore, CYP27B1 level was obtained using *Microplate Reader Biorad* Machine *model 680* with *software Microplate Manager ver. 5.2.1.* and measured using the ELISA method. The level of 25(OH)D_3_ was classified into deficiency (<20 ng/ml) and normal (≥20 ng/ml)^(12)^.

This study was conducted according to the guidelines laid down in the Declaration of Helsinki, and all procedures involving human subjects/patients were approved by the Research Ethics Committee of Faculty of Medicine, Universitas Indonesia with ethical clearance number LB.02.01/X.2/179/2016. Written informed consent was obtained from all subjects, before the study is started.

Collected data were then analysed using SPSS for Macintosh ver. 20. Characteristics of patients in the form of sociodemographic and clinicopathologically were analysed descriptively. Comparative and correlative analysis was done using unpaired *T*-test and Pearson for normally distributed data, also Mann–Whitney and Spearman for non-normally distributed data. This study used 5 % error bound and 95 % confidence interval limit, power of the test considered to be 90 %.

## Results

A total of sixty patients met the inclusion criteria and had been further analysed. Univariate test was performed to assess the general characteristics of the study subjects’ socio-demographic and clinicopathologic variables (Table 1).

**Table 1.** Clinical characteristics of subjects

Characteristics	Preterm (*n* 30)	*n* (%)	Term (*n* 30)	*n* (%)	*P*
Age (years)	28⋅27 ± 7⋅49		29⋅9 ± 5⋅53		0⋅341^a^
Gestational weeks	32 ± 3⋅0		38 ± 2⋅1		<0⋅001^a^
BMI (kg/m^2^)					
Pregnancy	22⋅78 ± 5⋅57		23⋅3 ± 3⋅61		0⋅339^a^
Underweight		8 (61⋅5 %)		5 (38⋅5 %)	
Normal weight		14 (42⋅4 %)		19 (57⋅6 %)	
Overweight		4 (80 %)		1 (20 %)	
Obese		4 (44⋅4 %)		5 (55⋅6 %)	
Birth	26⋅74 ± 5⋅76		28⋅75 ± 3⋅66		0⋅114^a^
Educational level					
Primary School		2 (100 %)		0 (0 %)	0⋅790^a^
Junior High School		7 (64⋅6 %)		4 (36⋅4 %)	
Senior High School		17 (45⋅9 %)		20 (54⋅1 %)	
Diploma		1 (33⋅3 %)		2 (66⋅7 %)	
Undergraduate		3 (42⋅9 %)		4 (57⋅1 %)	
Parity	0 (0–5)		1 (0–3)		0⋅240^b^
Birth weight					<0⋅001^a^
<2⋅500 g		27 (100 %)		0 (0 %)	
≥2⋅500 g		3 (9⋅1 %)		30 (90⋅9 %)	
Delivery method					0⋅004^a^
Vaginal		23 (65⋅7 %)		12 (34⋅3 %)	
Abdominal		7 (28 %)		18 (72⋅0 %)	

Data presented in Mean ± sd or Median (IQR).

a Unpaired *T*-test.

b Mann–Whitney.

Vitamin D status on preterm and term subjects were obtained and compared. Maternal serum 25(OH)D_3_ was classified into deficiency group (<20 ng/ml) and normal group (≥20 ng/ml). Results of this study can be found in Table 2.

**Table 2.** Vitamin D status of subjects

Variables	Preterm (*n* 30)	*n* (%)	Term (*n* 30)	*n* (%)	*P*
25(OH)D_3_ serum (ng/ml)	14⋅5 (5–53)		15⋅5 (5⋅3–33)		0⋅574^a^
Deficiency (<20 ng/ml)		20 (47⋅6 %)		22 (52⋅4 %)	0⋅573^a^
Normal (≥20 ng/ml)		10 (55⋅6 %)		8 (44⋅4 %)	
1,25(OH)_2_D_3_ serum (pg/ml)	62⋅9 (6–138⋅8)		75⋅5 (26⋅6–128⋅8)		0⋅214^a^
25(OH)D_3_ placental (ng/g)	21⋅5 (5–101)		44⋅0 (14–153)		0⋅001^a^
1,25(OH)_2_D_3_ placental (pg/g)	4⋅57 (0⋅24–12⋅23)		5⋅15 (1⋅46–18⋅95)		0⋅287^a^
1α-Hydroxylase (CYP27B1) (ng/mg)	0⋅007 (0⋅003–0⋅018)		0⋅008 (0⋅002–0⋅019)		0⋅755^a^
CYP27B1:25(OH)D_3_ placenta ratio (x 10^−3^)	0⋅35 (0⋅02–20⋅0)		0⋅17 (0⋅01–11⋅88)		0⋅016^a^
1,25(OH)D_3_:25(OH)D_3_ placenta ratio	0⋅175 (0⋅20–0⋅64)		0⋅089 (0⋅024–0⋅824)		0⋅071^a^

Data presented in Median (IQR).

a Mann–Whitney.

Furthermore, in order to determine the correlation between different vitamin D components, the correlation study was done to all variables. Significant correlation can be found on CYP27B1 with placental 25(OH)D_3_ (*r* −0⋅481, *P* < 0⋅001) and placental 1,25(OH)D_3_ (*r* −0⋅365, *P* = 0⋅004). Meanwhile, there was no correlation between other vitamin D components (*P* > 0⋅005).

## Discussion

The vitamin D derivate status was suspected to be less with preterm birth, and our study depicts the same result. We found that the median of 25(OH)D_3_ serum level in all subjects was 15 ng/ml, with the preterm subject had 1 ng/ml less than control. These results are lower than the average obtained in previous studies in the Southeast Asian region, with a range of 20–52 ng/ml^(10)^. Not statistically significant differences in 25(OH)D_3_ levels between preterm and term delivery also found in the previous study by Irwinda *et al.*^(8)^. These findings were found in contrary with another study conducted in China in 2013, which stated that mothers with serum 25(OH)D_3_ levels below 25 ng/ml had a significantly higher risk of experiencing preterm birth^(12)^. Another study also mentioned 25(OH)D_3_ serum >20 ng/ml has a protective effect against preterm birth^(13)^. In addition, significant differences found in placental 25(OH)D_3_ level (*P* = 0⋅001). This result is consistent with previous research in Jakarta^(8)^. Another study also showed a trend of significant increases in 25(OH)D_3_ level per trimester^(14)^. This data supported the anti-inflammatory effect as well as an immune system regulator of 25(OH)D_3_ in maternal serum and placenta, which may prevent preterm birth and preeclampsia^(6,9,13)^.

There were no significant differences levels of 1,25(OH_2_)D_3_ placenta between the two groups (*P* > 0⋅05). These results represent that there is no difference in the active form of vitamin D in placenta between preterm and term, but the lower inactive form of vitamin D was found in preterm birth, meaning that the process of active form was already converted in preterm birth, with a low reservoir level.

Moreover, no significant differences found in the CYP27B1 level, with placental CYP27B1 and 25(OH)D_3_ ratio is higher in preterm birth. Furthermore, the 1,25(OH)_2_D_3_–25(OH)D_3_ ratio in preterm patients was clinically higher, *albeit* statistically insignificant (*P* > 0⋅05). These results showed that in preterm labour, metabolically inactive 25(OH)D_3_ is broken down at a higher rate than in term labour. These findings are also similar to previous studies assessing the expression of CYP27B1 mRNA in rat placenta, which found no differences between term and preterm birth groups, even after vitamin D supplementation^(15)^. The previous study by Noyola-Martinez *et al.* also found that CYP27B1 expression would increase in the presence of some pro-inflammatory cytokines in trophoblast such as TNF-α, IFN-γ IL-6 and IL-1β^(16)^. This study also found a significant negative correlation between CYP27B1 and 25(OH)D_3_ and 1,25(OH)_2_D_3_ placenta. However, a stronger negative correlation was found between CYP27B1 and 25(OH)D_3_, in accordance with the function of CYP27B1 to change the form of inactive vitamin D to become active.

To our knowledge, this is the first study to directly compare different vitamin D status in serum and placenta of preterm and term women in Indonesia. However, no record of dietary intake and sun exposure during their pregnancy may become the limitation as it could interfere with the result of their vitamin D status. Regarding the method used to measure the level of CYP27B1 and 25(OH)D_3_ and 1,25(OH)_2_D_3_, the use of DiaSorin LIAISON showed the best characteristics among others for automated 25OH-D immunoassays. However, LCMS/MS isotope dilution still can be considered as the gold standard for small molecules analytic measurement. We also followed the Vitamin D council and Institute of Medicine (IOM) for the threshold for vitamin D deficiency (<20 ng/ml). This may differ from other studies using a higher threshold for categorising vitamin D deficiency. We used this as it has been used in many associations in society, and this number believed to already have a health impact, particularly skeletal health.

In conclusion, lower placental 25(OH)D_3_ status and higher placental CYP27B1 and 25(OH)D_3_ ratio was obtained in subjects with preterm compared to term birth.
